# P29 Outcomes by baseline pathogen and susceptibility in the ReSTORE Phase 3 trial of rezafungin once weekly compared with caspofungin once daily in patients with candidaemia and/or invasive candidiasis

**DOI:** 10.1093/jacamr/dlad066.033

**Published:** 2023-06-14

**Authors:** G R Thompson, A Soriano, O A Cornely, B J Kullberg, M Kollef, J A Vazquez, A F Das, J B Locke, T Sandison, P G Pappas

**Affiliations:** University of California Davis Medical Center, Sacramento, CA, USA; Hospital Clínic de Barcelona, Barcelona, Spain; University of Cologne, Faculty of Medicine and University Hospital Cologne, Cologne, Germany; Radboud University Medical Center, Nijmegen, the Netherlands; Washington University, St Louis, MO, USA; Augusta University, Augusta, GA, USA; Cidara Therapeutics Inc., San Diego, CA, USA; Cidara Therapeutics Inc., San Diego, CA, USA; Cidara Therapeutics Inc., San Diego, CA, USA; University of Alabama at Birmingham, Birmingham, AL, USA

## Abstract

**Background:**

Rezafungin is a next-generation, once-weekly echinocandin in development for treatment of candidaemia and invasive candidiasis (IC), and for prevention of invasive fungal diseases caused by *Candida*, *Aspergillus*, and *Pneumocystis* in allogeneic blood and marrow transplant recipients (Figure 1). ReSTORE (NCT03667690) is a global, double-blind, double-dummy, 1:1 randomized, controlled, Phase 3 non-inferiority trial that evaluated the efficacy and safety of rezafungin once weekly (QWk) versus caspofungin once daily (QD) in patients with candidaemia and/or IC. This analysis of the completed ReSTORE trial was conducted to evaluate outcomes by baseline pathogen and susceptibility.

**Methods:**

In ReSTORE, adults (≥18 y) with systemic signs and mycological confirmation of candidaemia and/or IC received either rezafungin QWk (400 mg Week 1, then 200 mg QWk) or caspofungin QD for ≥14 days (up to 4 weeks) with optional oral fluconazole step-down in the caspofungin arm. The primary endpoints were global cure at day (D)14 (per Data Review Committee confirmation of investigator-assessed clinical cure [and radiological cure for IC) + mycological eradication]) and all-cause mortality (ACM) at D30 (Figure 2). Secondary endpoints included mycological eradication at D14. For this analysis, D14 global cure and mycological eradication by treatment group were analysed by *Candida* species and in vitro susceptibility at baseline (CLSI broth microdilution MIC values; M27 Ed4) (Figure 3).

**Results:**

A total of 204 *Candida* isolates were recovered in 187 patients across both treatment groups. Of the 204 isolates, *Candida albicans* was the most common species, followed by *Candida glabrata*, *Candida tropicalis*, and *Candida parapsilosis*; 61% of all baseline isolates were non-albicans *Candida* (Figure 3). The rates of D14 global cure and mycological eradication by pathogen are shown in Tables 1 and 2. Overall, outcomes by *Candida* species and MIC did not appear to be affected by MIC values for either rezafungin or caspofungin (Table 3).

**Conclusions:**

Rezafungin was efficacious across multiple *Candida* species in the Phase 3 ReSTORE trial that demonstrated non-inferiority of rezafungin to caspofungin. There was no clear correlation between increased MIC values and clinical outcomes.

**Figure 1.**
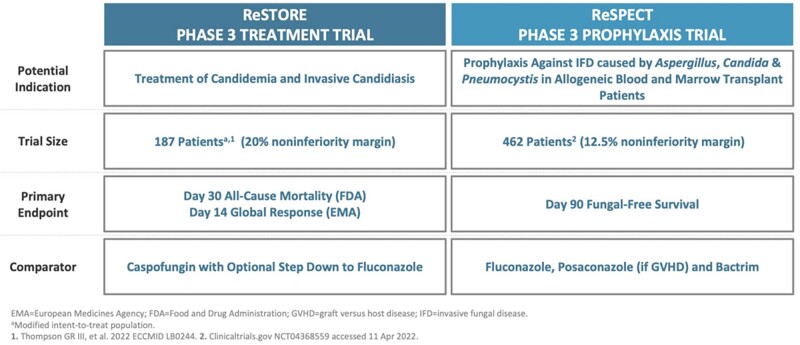
Rezafungin Phase 3 program.

**Figure 2.**
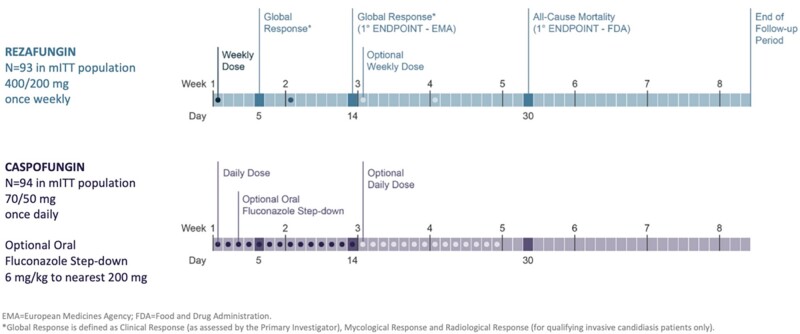
Study design of ReSTORE (NCT03667690).

**Figure 3.**
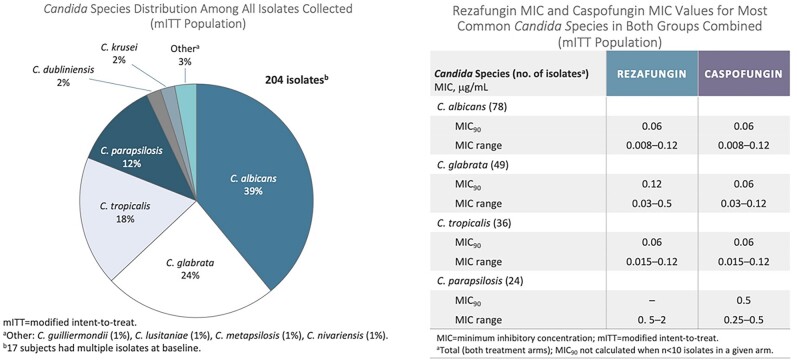
Candida species distribution and susceptibility at baseline in ReSTORE.

**Table 1.** Global response of cure at day 14 by most frequently isolated *Candida* species at baseline by treatment group (mITT)

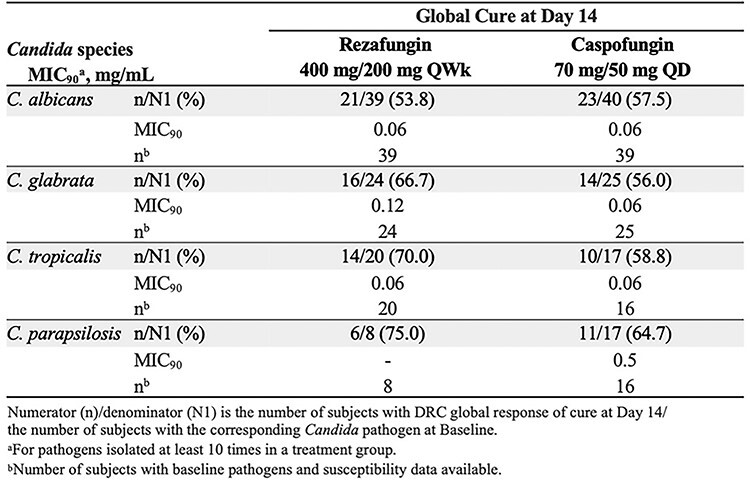

**Table 2.** Mycological response of eradication at day 14 by most frequently isolated *Candida* species at baseline by treatment group

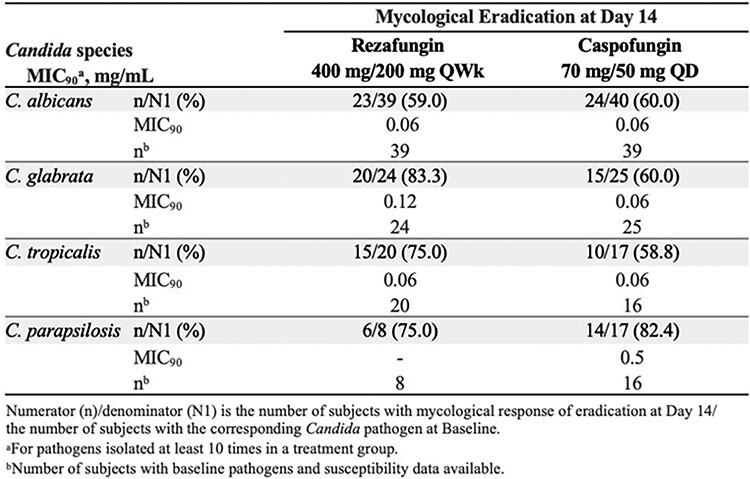

**Table 3.** Outcomes at day 14 by CLSI MIC values for most frequently isolated Candida species at baseline, by treatment group (mITT)

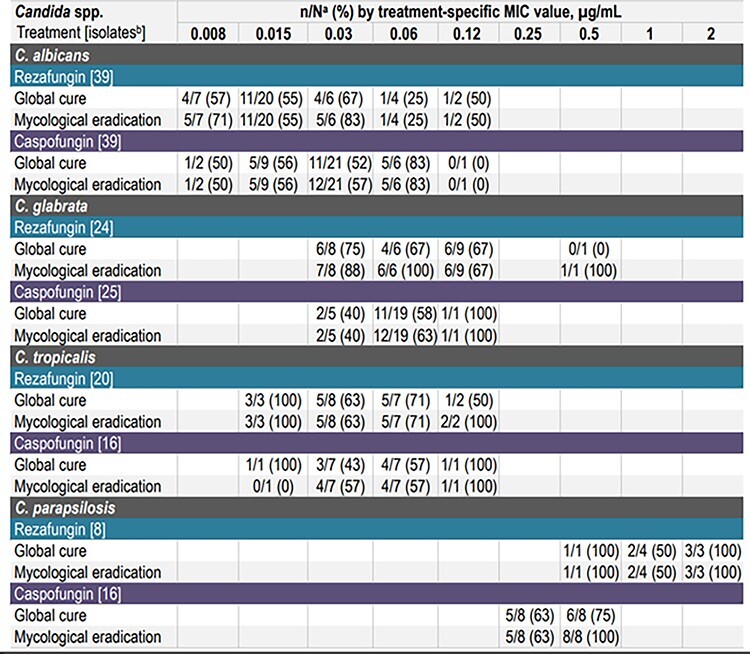

^a^Numerator/denominator is the number of subjects with listed response at day 14/the number of subjects with the corresponding *Candida* pathogen and MIC value for the respective treatment, as indicated by headers, at baseline. Not all isolates had MIC data.

